# Implementation of a Multimodal Mobile System for Point-of-Sale Surveillance: Lessons Learned From Case Studies in Washington, DC, and New York City

**DOI:** 10.2196/publichealth.4191

**Published:** 2015-11-26

**Authors:** Jennifer Cantrell, Ollie Ganz, Vinu Ilakkuvan, Michael Tacelosky, Jennifer Kreslake, Joyce Moon-Howard, Angela Aidala, Donna Vallone, Andrew Anesetti-Rothermel, Thomas R Kirchner

**Affiliations:** ^1^ Truth Initiative Evaluation Science and Research Washington, DC United States; ^2^ Department of Health, Behavior, and Society Johns Hopkins Bloomberg School of Public Health Baltimore, MD United States; ^3^ Department of Prevention and Community Health George Washington University Milken Institute School of Public Health Washington, DC United States; ^4^ Survos LLC Washington, DC United States; ^5^ College of Global Public Health New York University New York, NY United States; ^6^ Department of Sociomedical Sciences Columbia University Mailman School of Public Health New York, NY United States; ^7^ Steven A Schroeder Institute for Tobacco Research and Policy Studies Truth Initiative Washington, DC United States; ^8^ Department of Oncology Lombardi Comprehensive Cancer Center Georgetown University Medical Center Washington, NY United States

**Keywords:** mobile technology, public health surveillance, tobacco, point-of-sale, implementation, tobacco industry advertising, marketing

## Abstract

**Background:**

In tobacco control and other fields, point-of-sale surveillance of the retail environment is critical for understanding industry marketing of products and informing public health practice. Innovations in mobile technology can improve existing, paper-based surveillance methods, yet few studies describe in detail how to operationalize the use of technology in public health surveillance.

**Objective:**

The aims of this paper are to share implementation strategies and lessons learned from 2 tobacco, point-of-sale surveillance projects to inform and prepare public health researchers and practitioners to implement new mobile technologies in retail point-of-sale surveillance systems.

**Methods:**

From 2011 to 2013, 2 point-of-sale surveillance pilot projects were conducted in Washington, DC, and New York, New York, to capture information about the tobacco retail environment and test the feasibility of a multimodal mobile data collection system, which included capabilities for audio or video recording data, electronic photographs, electronic location data, and a centralized back-end server and dashboard. We established a preimplementation field testing process for both projects, which involved a series of rapid and iterative tests to inform decisions and establish protocols around key components of the project.

**Results:**

Important components of field testing included choosing a mobile phone that met project criteria, establishing an efficient workflow and accessible user interfaces for each component of the system, training and providing technical support to fieldworkers, and developing processes to integrate data from multiple sources into back-end systems that can be utilized in real-time.

**Conclusions:**

A well-planned implementation process is critical for successful use and performance of multimodal mobile surveillance systems. Guidelines for implementation include (1) the need to establish and allow time for an iterative testing framework for resolving technical and logistical challenges; (2) developing a streamlined workflow and user-friendly interfaces for data collection; (3) allowing for ongoing communication, feedback, and technology-related skill-building among all staff; and (4) supporting infrastructure for back-end data systems. Although mobile technologies are evolving rapidly, lessons learned from these case studies are essential for ensuring that the many benefits of new mobile systems for rapid point-of-sale surveillance are fully realized.

## Introduction

Public health surveillance is necessary for collecting health data and for planning and evaluating programs and policies [1]. In tobacco control and other fields, point-of-sale surveillance of the retail environment is critical for understanding industry marketing of products and informing public health practice [2]. Innovations in mobile technologies provide opportunities to improve existing surveillance methods. Recent studies comparing mobile versus paper-based data collection for surveillance have identified several benefits of mobile technologies, including reduced data loss [3], real-time quality control [3], rapid use of data [3,4], and reduced costs [4]. Mobile phones have the added benefit of being less conspicuous than paper surveys [4]—an important consideration in retail surveillance.

Previous retail assessments of tobacco marketing and related public health topics such as alcohol marketing and food availability have traditionally involved paper surveys completed by trained data collectors within a sample of representative stores [5-9]. More recent studies mention other modes of data collection for tobacco point-of-sale assessment [10-13], but few report their methodology with any detail [6] and none have described the challenges and potential solutions for implementing multimodal mobile systems that incorporate data from different sources. A detailed and well-planned implementation process is critical to ensuring surveillance systems gather up-to-date, accurate information quickly and efficiently, which is especially critical for low-resource environments.

This paper aims to address this gap in the literature by sharing implementation lessons from 2 point-of-sale surveillance projects, with the objective of preparing public health practitioners for the process of implementing multimodal systems using mobile technologies for point-of-sale marketing assessments. First, we describe the background of each project and the mobile systems and tools used. We then describe the preimplementation field testing process, providing examples of challenges and solutions to issues confronted during testing. We summarize by providing lessons learned for implementing mobile technologies for point-of-sale surveillance.

### Project Descriptions

From August 2011 to May 2013, our research group conducted 2 point-of-sale surveillance projects in Washington, DC, and New York City. The goals of these projects were (1) to capture point-of-sale marketing information to educate communities about tobacco marketing in their neighborhoods and inform policy and (2) to assess the feasibility of multimodal mobile data collection tools for point-of-sale surveillance.

#### Washington, DC, Point-of-Sale Surveillance Project

The Washington, DC, project was funded by the District of Columbia (DC) Department of Health and Truth Initiative (known as the “American Legacy Foundation” at the time of this project) and ran from August 2011 to March 2012. This project was a traditional research study, in which researchers hired and trained paid professional fieldworkers to collect data on tobacco advertising in all outlets licensed to sell tobacco (n=1061) in DC, a midsized urban city of approximately 650,000 people, with a large African-American population. More details on the study can be found elsewhere [14-17].

#### New York City Point-of-Sale Surveillance Project

The New York City (NYC) study was funded by Truth Initiative and was conducted from February to May 2013. This study was a community-based participatory research project and involved a comprehensive survey of all outlets within selected census tracts in Central Harlem, a predominantly low- to moderate-income, African-American community with a total of approximately 156 outlets that serve a local population of 165,000 people over a 2.1-mi^2^ area [18-20]. The study included the formation of an academic-community partnership between researchers from Truth Initiative, Columbia University’s Mailman School of Public Health, and community representatives with experience in tobacco control work in Harlem. Local students recruited through a nonprofit Boys & Girls Club were paid a small stipend for conducting data collection. More details on this study can be found elsewhere [21].

Both projects were designed to include some or all of the following components:

1. *Audio or voice recording data:* Responses to survey questions (by selecting or typing a response or by voice recording) on tobacco advertising on the exterior and/or interior of outlets;
2. *Electronic photographs:* Images of tobacco advertisements in outlets;
3. *Electronic location data:* Information about the outlet, such as the store name, address, and/or GPS coordinates.

### Surveillance System

Prior to the commencement of the DC and NYC projects, the authors worked with an information technology service provider to develop a flexible multimodal surveillance system to collect data through digital, or nonpaper-based, methods. The system was based on a server-client model in which data could be entered via a mobile device or computer (ie, acting as the “client”) and communicate directly and in real-time with the remote server. Data were transmitted via a wireless local area network or via a mobile phone network.

### Software

Software allowed for data collection via mobile technologies utilizing text messaging, email, GPS technologies, and phone-based interactive voice response (IVR), a technology that allows a computer to interact with humans through the use of voice and touchtone input via keypad. The IVR software was written in PHP (a Web-based programming language) and integrated with a telephone provider (Voxeo), communicating via the VoiceXML protocol, which is designed specifically to enable IVR and remote computer systems to exchange data. In addition, a test version of a custom mobile application functional for both iOS and Android operating systems was developed prior to the NYC project. This app was not available for the DC project.

### Server and Dashboard

All data from the field were automatically sent to and stored in a secure server. The server provided authorized users with access to a Web interface that contained tools for visualizing and managing data.

## Methods

### Data Collection Modes

For each project, we chose tools from the multimodal system and software based on project needs, software availability at the time, fieldworker expertise, and the environment in which the project was implemented. For the DC study, primary data collection modes included an IVR-programmed survey taken using any mobile phone and photographs taken with a mobile phone with a built-in camera and emailing capabilities. For NYC, tools included an IVR-programmed survey accessed via mobile phone and the aforementioned mobile app on a smart phone used to take photographs.

A preimplementation field period involved conducting a series of iterative tests to inform decisions and establish protocols around the components of the project, described below. The DC field testing period lasted 7 weeks and included field testing, survey instrument development, and reliability testing. Applying lessons learned in DC, our field testing period for NYC was approximately 2 weeks.

### Mobile Phone Selection

Field tests involved photographic or survey data collection at a limited number of stores using a specific mobile phone, with fieldworkers reporting on problems encountered in the field with the device, using that feedback to inform the next series of tests, and repeating the process until final mobile phone criteria were established.

### Workflow and User Interface

For both projects, the workflow involved unobtrusively taking photographs and collecting survey data on tobacco advertising for each store exterior and interior. The user interface in this study referred to the fieldworker interaction with the IVR survey, phone cameras, phone email, and mobile app. Different workflows were tested along with varying aspects of the user interface until the most efficient process was established.

For fieldworker training and technical support, we allotted time for initial training on the specifics of tobacco advertising data collection as well as practicing with the technologies, testing the workflow and interface in the field, and establishing a feedback loop between fieldworkers and researchers.

For the back-end data system, researchers and fieldworkers tested different strategies for linking survey, photo, and location data collected from various modes to an individual store, with different solutions developed for each project. As part of the linking and geocoding process, we obtained lists of all businesses licensed to sell tobacco in the area from the city governments, which included business names and addresses. We geocoded addresses for each study utilizing the Master Repository Geocoder from the DC Office of the Chief Technology Officer [22], a free tool available for addresses within the District. For NYC, we utilized ArcGIS Online geocoding services [23], which were free at the time. The research team also worked with the technologist to develop a custom-made, secure online website that allowed for real-time posting and monitoring of data.

To come to decisions on key issues, the full interdisciplinary team—including researchers, project managers, technologists, and field staff—met formally 1-2 times per week to discuss relevant components of the project, identify the specific challenges and decisions to be addressed, and work through each issue utilizing a group consensus-based approach. Relevant materials were provided before or during the meeting. During the user interface and website development phases, the technologist team provided versions of the survey via IVR or updates to the website to be tested by team members and subsequently discussed during meetings to resolve major issues and decide on next steps. Additional discussions were held on a Web forum established during the pilot process to resolve field testing issues (described below) and over email. Smaller or more urgent decisions were often made on a daily basis using these electronic channels or daily debriefs with fieldworkers. Preliminary lessons learned were decided upon by the team and based on the first DC project; they were then further refined after the NYC project for the purposes of this paper.

## Results

Below we discuss the main challenges and solutions that resulted from the field testing process, including examples to illustrate how technical and other challenges were resolved for project implementation.

### Choosing a Mobile Phone

The choice of a mobile phone will facilitate or hinder usability, visibility, and quality of data collection, thus criteria for phone selection should be established based on the type of data to be collected and following a series of field tests. For both projects, most mobile phones with cellular service could be used for IVR survey data collection. However, the collection of multiple high-quality photographs and linking photos with survey and store identification data required further criteria for mobile phone selection. Criteria established included the following:

1. *Wireless technologies:* A minimum of 3G cellular connectivity was required. For the NYC project, phones also required Wi-Fi connectivity so fieldworkers could quickly upload photos to the server from the mobile app via a wireless connection as it was often too slow to upload photos over the cellular network.
2. *Operating system:* Either an Android or iOS operating system was required for compatibility with apps that were in development or in use during the projects.
3. *Camera:* A rear-facing camera with a minimum of 5-8 megapixels was required. A higher number of megapixels indicates higher quality photographs. Camera features should allow for the ability to disable the flash and shutter noise, which is essential for being unobtrusive while taking photos in the retail environment. The camera must also allow for geotagging of photos when GPS is turned on.
4. *Display:* The display technology of the phone had to allow for high resolution visibility outdoors and in direct sunlight, preferably with a Super AMOLED touchscreen.
5. *Battery life:* Phone battery life for talk time had to extend through the length of at least one field shift, which was usually 4-6 hours long.
6. *Global positioning system (GPS):* The phone required GPS-enabled receiving capability.
7. *Other features:* Phone had to have a headphone jack for listening to the IVR survey and reducing background noise.

Based on the aforementioned criteria, we chose the Samsung Focus in 2011 for the DC project and the Samsung Galaxy S II 4G in 2012 for the NYC project.

### Workflow and Front-End User Interface

**Figure 1:**
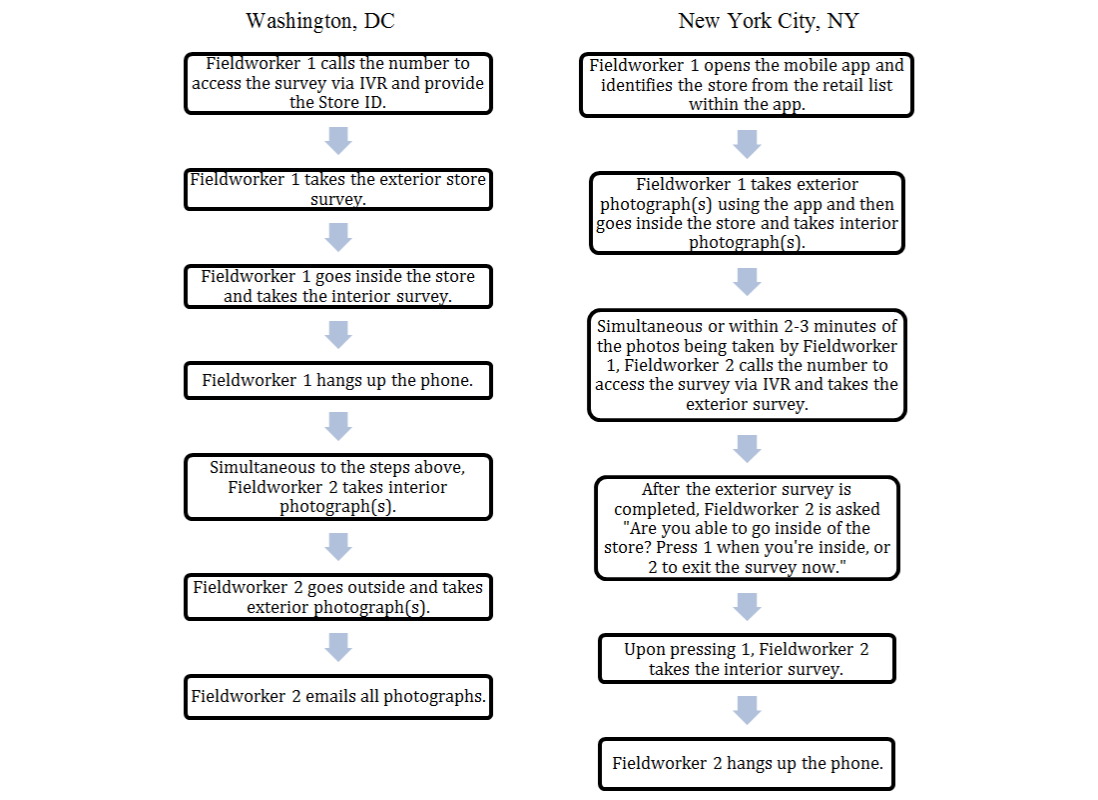
Final optimized workflow for DC and NYC.

The field testing process demonstrated that utilizing fieldworker pairs to survey stores was needed for safety purposes and to optimize workflow. Streamlining the field data collection necessitated that the fieldworkers share tasks at each store and required allowing for time and flexibility during data collection to respond to delays or unexpected events. Figure 1 outlines the workflow used in DC and NYC. Based on lessons learned in DC, we added questions to the survey for NYC that allowed fieldworkers time to pause the IVR survey when needed to move around naturally (ie, from the exterior of the store to the interior).

#### User Interface: IVR Survey

In developing a streamlined user interface, we established 3 key guiding principles: simplicity, accuracy, and flexibility. Fieldworkers tested different stages of the user interface in the field and evaluated the interaction in light of these principles. The most challenging interface was the user’s interaction with the IVR for the purpose of taking the survey. Audio surveys taken via IVR take longer than pencil-and-paper surveys and make complicated survey questions difficult. IVR questions were tested in training sessions and in the field to identify improvements for shortening each question item’s stem and response categories and improving overall survey flow. Table 1 provides examples of the application of the 3 principles to resolve challenges with the IVR survey.

**Table 1:** Principles and examples for streamlining user interface components for the IVR interface.

Principle	Issues	Solutions
Simplicity	Audio surveys via IVR may take much longer than pen-and-paper surveys and can make complicated survey questions difficult.	Utilize clear and simple language for survey questions; allow for shortcuts and skip patterns where feasible.
		Ideally, keep survey instruments short (approximately 5-10 minutes) and focused on a limited number of surveillance topics. Construct question items to be concise and direct. For surveys in both cities, items are allowed for primarily dichotomous (Boolean) or multiple-choice response categories for ease of data entry, although some items allowed surveyors to enter data (such as counts and prices).
		Test questions via IVR to clarify and simplify each item’s question stem, response categories, and flow as experienced in the field.
Accuracy	Fieldworkers are speaking responses and cannot visibly see what is being recorded to check their entry, correct something they misspoke, or that the system misheard.	Incorporate a brief check after each question to assess whether the entry was entered correctly. Upon each answer, the system confirmed the response. For example, if a fieldworker entered “liquor” to the question “what type of store is this?,” the IVR system would immediately say, “You said ‘liquor.’ Is that correct?” If the fieldworker entered “yes,” they would move on to the next question but if they entered “no,” the IVR system would ask the question again and allow the fieldworker to enter the correct answer.
	Background noise makes it hard for fieldworkers to hear survey questions and can also trigger false responses on the IVR if the system mistakes background noise for a response.	Design system to repeat questions until a response is provided.
		Use headphones and the phone’s keypad to answer questions; mute phone in very loud areas.
Flexibility	With largely closed-ended responses, it was sometimes difficult to capture information on new brands appearing at the point-of-sale or other relevant commentary.	Allow surveyors to provide nonscripted information through IVR open-ended voice responses to specific questions, which may later be coded.
		Incorporate photos into the data collection process. Photos can provide qualitative data on product information that may not have been captured in the survey and can occasionally serve as a check on survey data.
	In the field, unexpected issues might arise, such as confrontation by a store employee, which require individuals to exit the store or hang up on the survey.	The survey should allow for fieldworkers to hang up midsurvey and pick up from the last section they had started without losing previous data entered for the store.
	Speaking responses into the phone to answer survey questions via IVR can call attention to fieldworkers, especially in small stores.	Fieldworkers should use headphones to listen to the survey and answer the questions using the phone’s keypad, which works for most questions, with the exception of open-ended questions.

#### User Interface: Photographs

**Figure 2:**
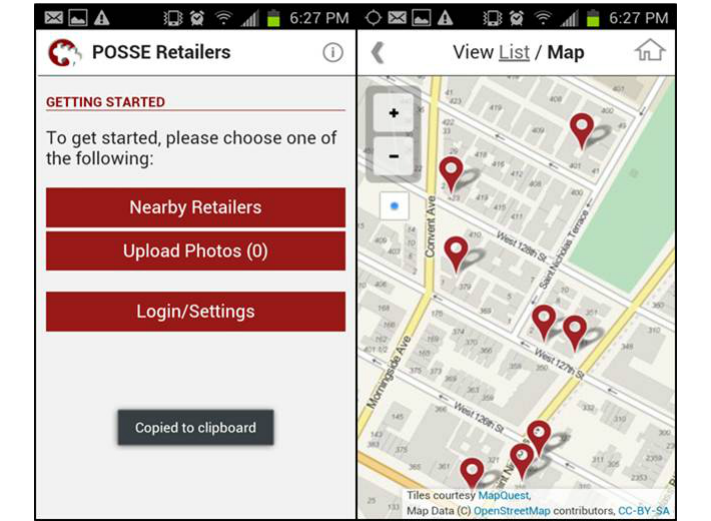
Mobile app for NYC (map view).

While the interface for taking and emailing photographs on a mobile phone is straightforward, these activities must be seamlessly integrated into a multicomponent, multimodal data collection workflow so as not to slow down the store assessment process. For the DC project, we established through field testing that only 1 exterior photograph of the main storefront and 1 interior photograph of a checkout countertop area were required. We encouraged fieldworkers to take additional photos to document tobacco advertising; in practice, however, fieldworkers could only email 1 photo at a time given the back-end need for linking photos to store IDs, which made the process time consuming and tedious. For the NYC project, we incorporated this learning from DC by building a basic mobile app that allowed fieldworkers to take and upload multiple photos for each store in a single step. Taking photographs and linking them to individual stores was streamlined within the app by allowing fieldworkers using GPS to view nearby stores in the “list” mode, which listed stores by proximity, or in the “map” mode, which displayed a map populated by nearby stores based on proximity (Figure 2). Fieldworkers could then select the store and take multiple photos, which were easily and automatically linked to each store.

### Fieldworker Training and Technical Support

Once fieldworkers were trained on the basics of tobacco advertising, they tested the system with the project manager and in pairs. We established a Web forum during testing and training so that fieldworkers could describe issues that arose in field tests and the team could view daily posts and decide on solutions to common problems. Once protocols were established, fieldworkers underwent a comprehensive technical training with the project manager (Textbox 1).

**Technology training protocol.**

- Conduct one-on-one instruction between project manager/trainer and fieldworker.
- Review how to use the IVR survey on project phones, how to access various project phone numbers, and how to turn on GPS tracking.
- In DC, review how to take appropriate photos with the project phone, attach the photo (or multiple photos) to an email, and send them to the designated project email addresses linked to the study database; in NYC, review how to use the application to identify stores and how to take and send photos.
- Practice taking the IVR survey in the office using photographs from the field to get comfortable with the survey flow.
- Project manager/trainer accompany each fieldworker independently into local neighborhood stores to practice using the technology in the “real world” setting of a retail outlet.
- After in-field training, project manager and fieldworker debrief to discuss challenges and strategies for how to deal with the technology in the retail environment.
- Provide booster trainings throughout project implementation for fieldworkers having difficulty with the technology.

We also established real-time technical support throughout implementation by ensuring a manager and/or technologist were on call when fieldworkers were working to resolve problems quickly. For example, during the DC project testing, the IVR provider would occasionally shut down. On these occasions, fieldworkers would contact the person on call, who could quickly resolve the issue by notifying the IVR provider and keeping fieldworkers updated.

### Back-End Data Systems

**Figure 3:**
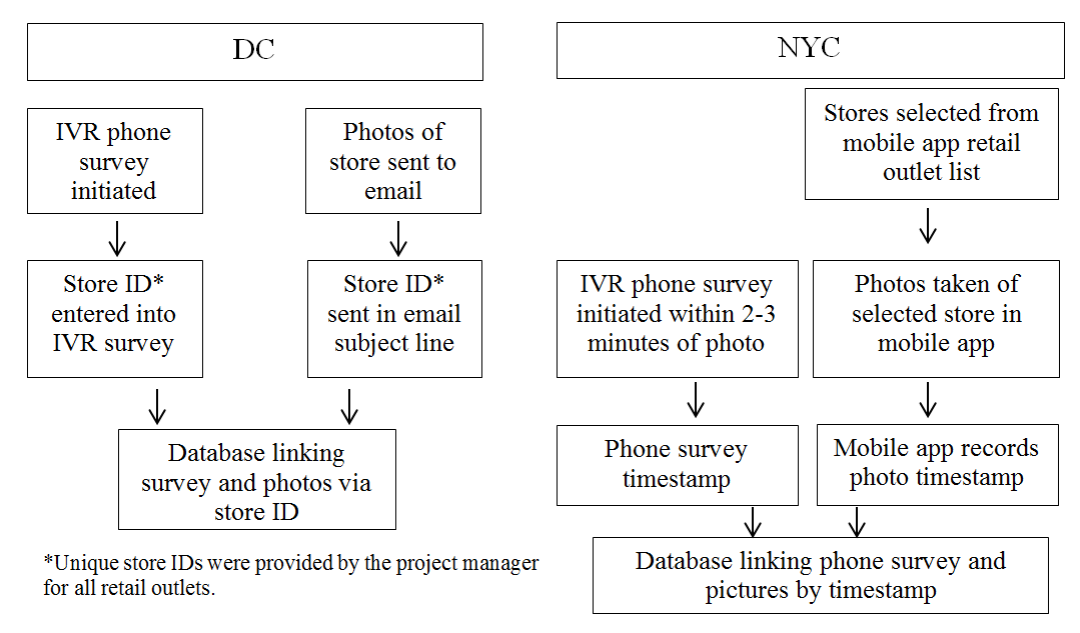
Linkage process for store survey and photo data by project.

To link store, survey, and photo data in the DC project, the project manager established store IDs linked to store names and addresses. Fieldworkers then entered the IDs when conducting store surveys or emailing photographs. For NYC, the consistent use of accurate store IDs proved challenging among the volunteer fieldworker staff. Because the photo data were linked to store names and addresses through the mobile app, we established a system of linking survey data from the phone surveys and photo data from the mobile app via a timestamp. Figure 3 provides a visual of the ID linking process for each project. Ideally, the linking of survey and photo data to store IDs would be automatic via the use of a mobile app that incorporated survey, photo, and location data collection linked to a more advanced, integrated data infrastructure than was available at the time of these studies.

The full utilization of GPS technologies was limited in each of these projects given the early stages of systems development. For both projects, we conducted geocoding using a geographic information system (GIS) based on store address via a somewhat onerous process that required downloading and cleaning files, uploading files into GIS systems for geocoding, and downloading and cleaning again. For both cities, we used a minimum matching score of 90% to batch geocode the addresses and manually geocoded any matches below 90%. In a later version of the back-end system that was developed subsequent to the DC and NYC projects, all data systems were streamlined so that different sources of data—particularly GPS data based on fieldworker routing—store IDs, and data collected were synchronized automatically.

**Figure 4:**
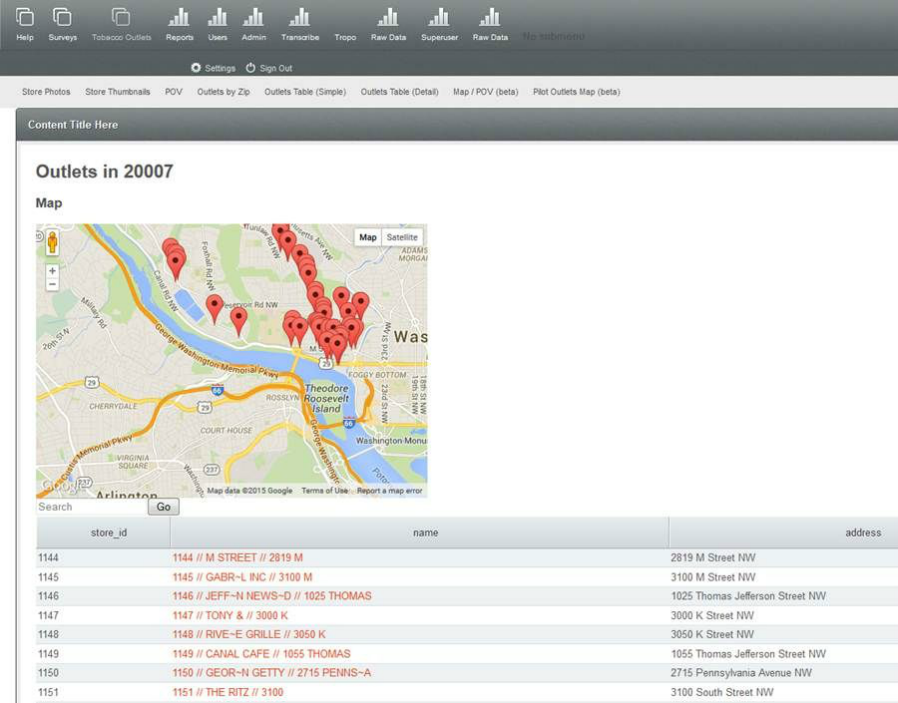
Back-end database with map of stores assessed by ZIP code.

**Figure 5:**
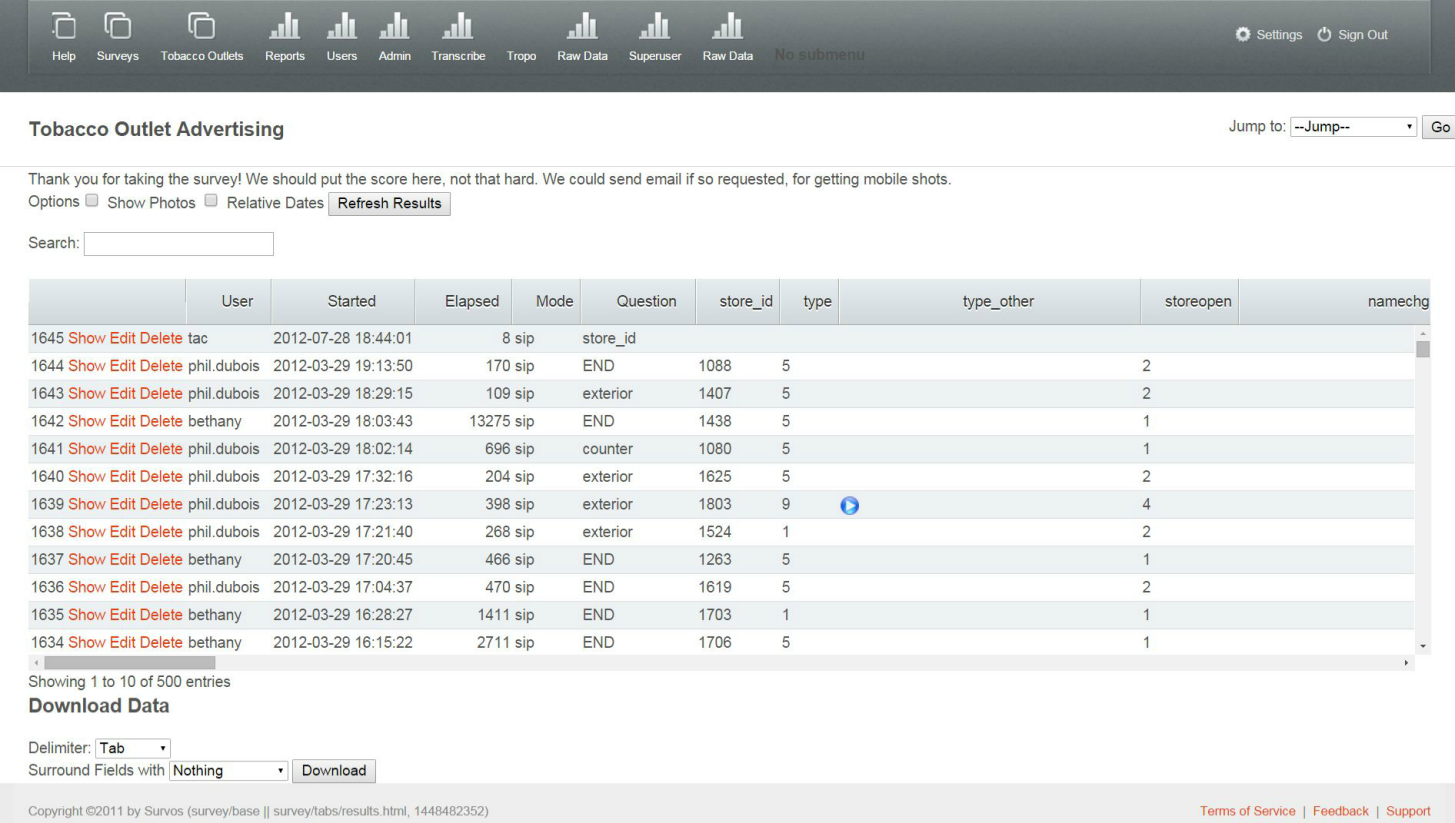
Back-End Database with Table of Individual Store Observations Updated in Real Time.

**Figure 6:**
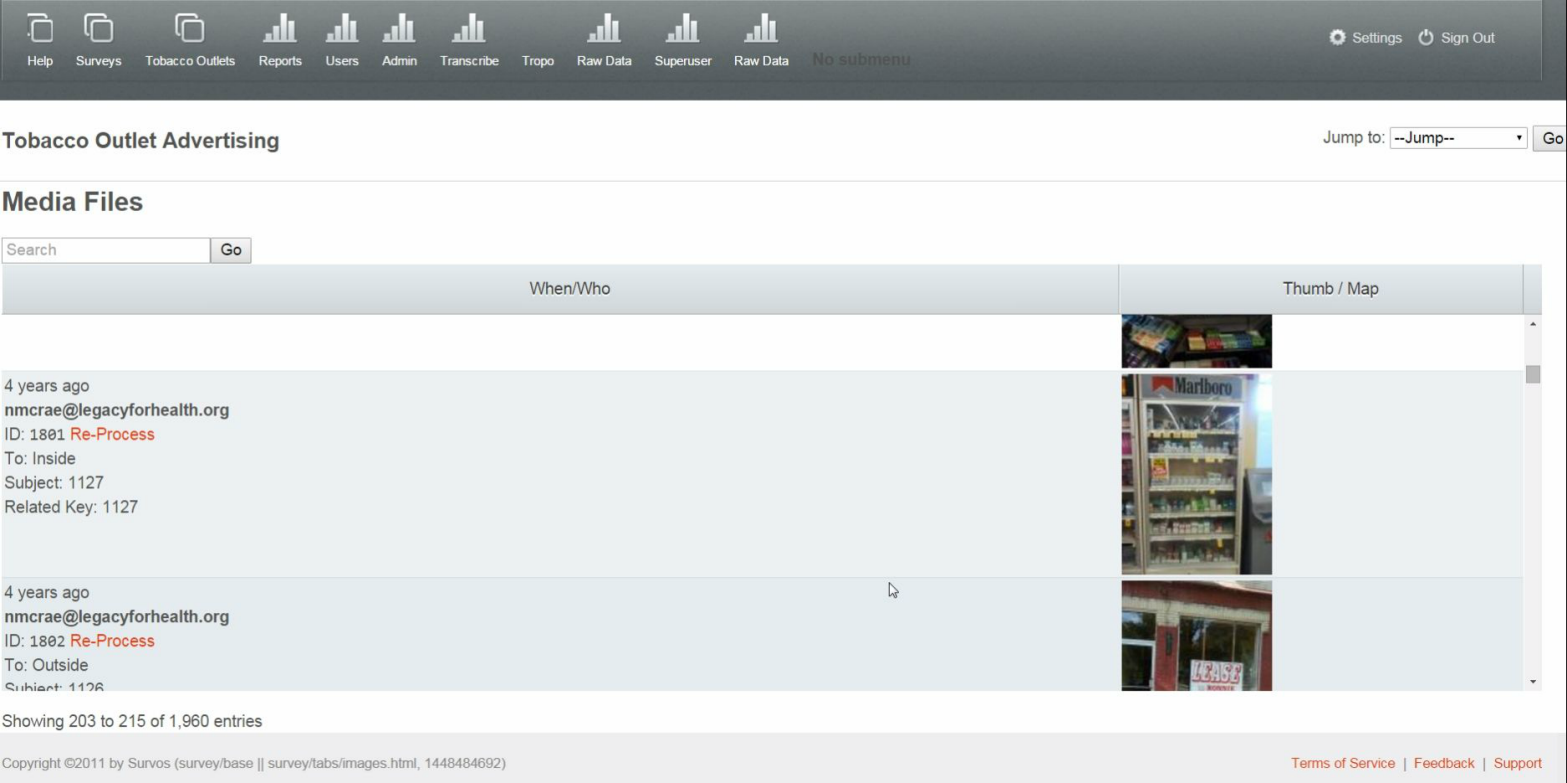
Back-End Database with Photographs from Data Collection.

**Figure 7:**
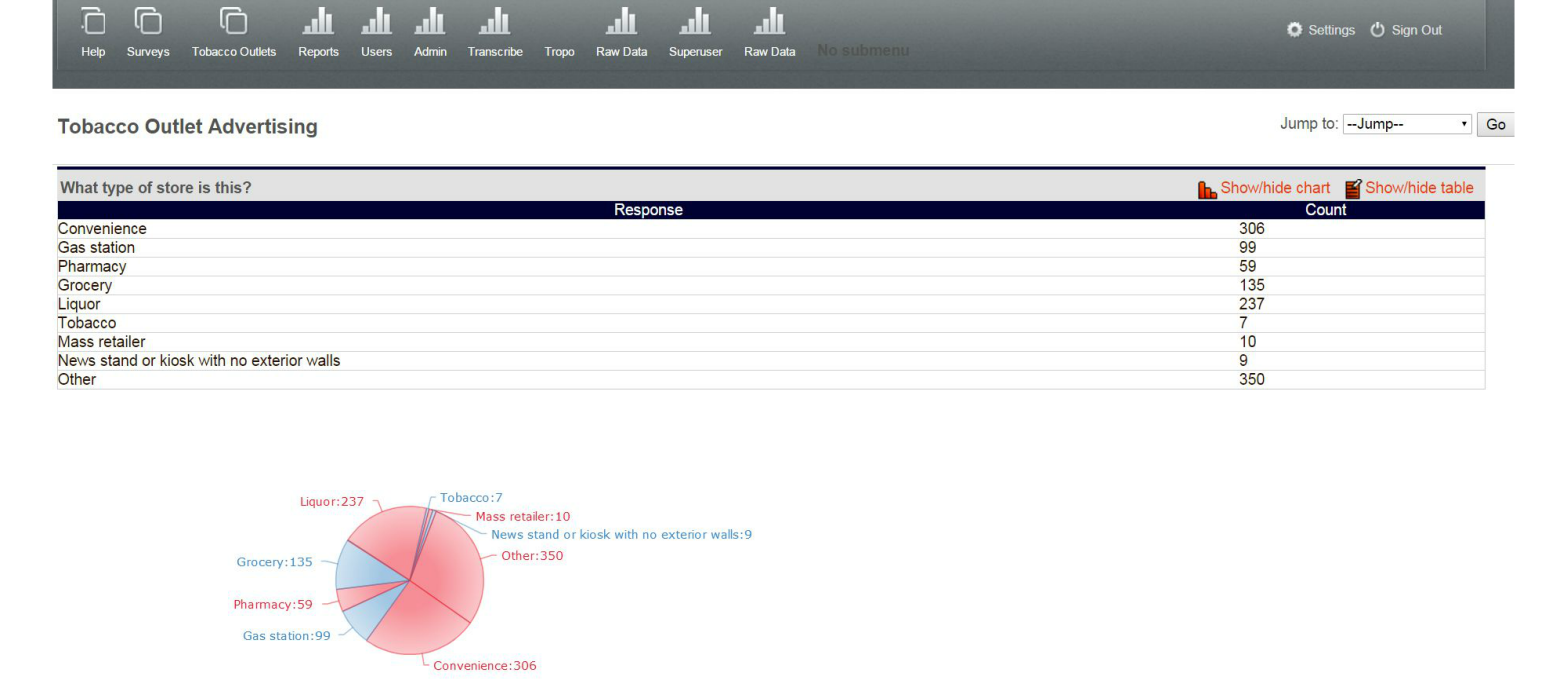
Back-End Database Summary Table and Pie Chart of Aggregated Variables.

To develop the website, we worked closely with the technologist, testing different aspects of the site to specify the components of a simple and interactive dashboard that allowed staff to monitor data collection in real-time and to access updated datasets. Components included: a map of stores assessed by ZIP code (Figure 4); a table of individual store observations from the IVR survey updated in real time (Figure 5); photographs with specified identifiers (Figure 6); visuals of aggregate data for each variable in the survey updated in real-time (Figure 7); a copy of the survey and a test version of the survey; a project management calendar; and the ability to export survey and photographic data in standard file formats, such as comma separated values and Microsoft Excel.

## Discussion

Multimodal mobile systems for point-of-sale surveillance present opportunities to collect, analyze, and disseminate retail data in real-time to researchers and community stakeholders. Yet implementation of seemingly straightforward tools requires that researchers and practitioners familiarize themselves with processes that are common to technology development and engineering [24-26]. Below we describe lessons learned from the DC and NYC projects that are applicable to mobile technology implementation projects for point-of-sale surveillance.

### Lesson 1: Implementation of Mobile Systems Requires an Iterative Testing Process

Implementation of mobile systems introduces new technical decisions that can be resolved via an iterative testing approach prior to project launch. We utilized quick, continuous, and repeated testing to ensure that the technology, data collection workflow, and user-interface were workable and user-friendly in different situations and for multiple users. This process was critical to identifying technical, individual, and environmental challenges that influenced the performance of fieldworkers and the accuracy of data collection. Studies examining the use of mobile phones for interventional purposes have also noted the need to incorporate rapid, repeated testing and development phases in pilot work [26,27].

Testing time and a budget should be built into a pilot field testing period, with more investment required early on and generally decreasing costs as teams learn from each project. Budgets for pilot field testing mobile data collection will vary by project and may include fieldworker costs (unless volunteers are used), mobile phone costs (ie, phones, cellular and/or wireless service), technologist costs if an outside technology provider is used, and costs for the project management or research team. For these studies, Truth Initiative funded a local technology provider for the initial development of the multimodal mobile system and for support during the field testing and implementation process. Open source software or other resources to develop and implement mobile data collection surveillance studies are also becoming increasingly available [28].

### Lesson 2: Workflow and User Interface Decisions Require Attention and Testing

Data collection workflow and the user interface take on increased importance with the use of new technologies for surveillance. Ease of use and usefulness to the end user is closely linked to the performance of a technology [29,30] and to effective implementation of mobile surveillance projects [31-34], thus it is critical to consider the experience of the primary users of the system. Therefore, as the main user of the system, we put the fieldworker at the center of the process for establishing the workflow protocol and streamlining the interface. This helped frame questions about what data to collect from a user-centered perspective of how the information could best be collected using the technology to ensure simplicity, accuracy, and flexibility.

### Lesson 3: Training and Implementation Require Close Communication, Feedback, and Ongoing Technical Assistance

Developing feedback loops for consistent communication between fieldworkers, researchers, and the technologist—along with rapid ongoing technical assistance—is critical during testing and deployment to quickly resolve problems and keep field staff committed to using the technology correctly [34-36]. This also assures researchers that data collection and quality will not be undermined by technical glitches. During the iterative testing process, the Web forum and daily debriefs with fieldworkers kept all staff up to date with technical, workflow, or other field problems and allowed for immediate problem solving. Thi*s* process served as a training ground for fieldworkers who were involved in the early stages of the project and, importantly, allowed for the development of knowledge and skills among all staff for quickly resolving technical glitches and moving the project forward.

### Lesson 4: Back-End Data Infrastructure and Automated Systems Require Development and Integration

Automatic collection of data from the field to a back-end server provides many potential benefits, including allowing managers to utilize real-time quality control while data is being collected to improve data accuracy and quickly resolve logistical difficulties. However, these tools require that systems are integrated and, ideally, automatically link different sources of data from the field together for real-time monitoring, integration with GIS systems, and analyses. This process takes time and planning to identify the key issues for each project for integrating data sources, linking data from multimodal mobile sources to store identifiers, ensuring GPS can be collected and used efficiently in GIS systems, and ensuring skilled staff or outside consultants are available to develop and maintain this automated back-end infrastructure. Careful planning for back-end systems can ensure that a key benefit of multimodal mobile surveillance—rapid, automated, and visualized data collection—can be fully utilized.

### Limitations

Our findings should be considered with the following limitations:

1. This study was not designed to examine the validity or reliability of mobile phone data collection for surveillance compared with more traditional paper-based methods, so it cannot be assumed these methods lead to improved data quality compared with paper surveys. We have, however, clarified some of the benefits of mobile data collection in comparison with paper surveys.
2. Fieldworkers and staff used their own phones in the early stages of testing until phone criteria were developed and phones were purchased. In projects where staff cannot use personal phones, field testing to establish mobile phone criteria would likely require additional funding or rely more on online research. Given that mobile technology is advancing at a rapid pace, projects that last more than 6 months to 1 year may need to update mobile phone hardware to take advantage of new mobile phone technologies as well as developments in software and automatic data integration, all of which will impact multiple components of a project.
3. Lists of licensed tobacco retail outlets in the geographic areas surveyed were available for these projects. Not all cities or states require or provide such lists, thus field surveying of stores may be necessary in some areas and may require additional strategies for identifying retail outlets and linking data from different sources to specific stores through mobile systems.

These projects were conducted in the United States, thus different or additional lessons may be applicable in international settings. Further, as technology continues to evolve, new lessons may apply.

### Conclusions

Mobile technologies may improve surveillance efforts and facilitate rapid policy responses to emerging tobacco products and industry marketing practices. Information on technology implementation in health care and health research is sparse, which may be one factor in the limited number of large-scale implementation efforts despite a growing literature indicating promising findings from technology and health pilot studies [37]. Implementing new technologies with an iterative testing framework that (1) ensures a smooth workflow and user-friendly interfaces; (2) allows for training, ongoing communication, and feedback among all staff; and (3) supports infrastructure for back-end data integration is critical for successful system performance.

This is the first study to examine implementation needs for conducting multimodal mobile tobacco retail surveillance. Although mobile technologies are evolving rapidly, these implementation lessons are relevant for those who want to harness the strengths of any new mobile system for point-of-sale surveillance or collection of other types of neighborhood observational data. Findings suggest the need for further research examining mobile surveillance implementation challenges, as well as funding for developing and implementing systems that allow public health practitioners and researchers to improve surveillance by taking advantage of the communications tools of the twenty-first century.

## Abbreviations

DC: District of Columbia
GIS: geographic information system
GPS: global positioning system
IVR: interactive voice response
NYC: New York City
